# Supplementary material for: The extracellular matrix regulates cortical layer dynamics and cross-columnar frequency integration in the auditory cortex
Source: Commun Biol. 2021 Mar 10;4:322. doi: 10.1038/s42003-021-01837-4 (PMC7946889; doi:10.1038/s42003-021-01837-4)
Supplement: Supplementary file 2 — Supplementary Information [file 42003_2021_1837_MOESM2_ESM.pdf]

Supplementary Information (El-Tabbal et al.)

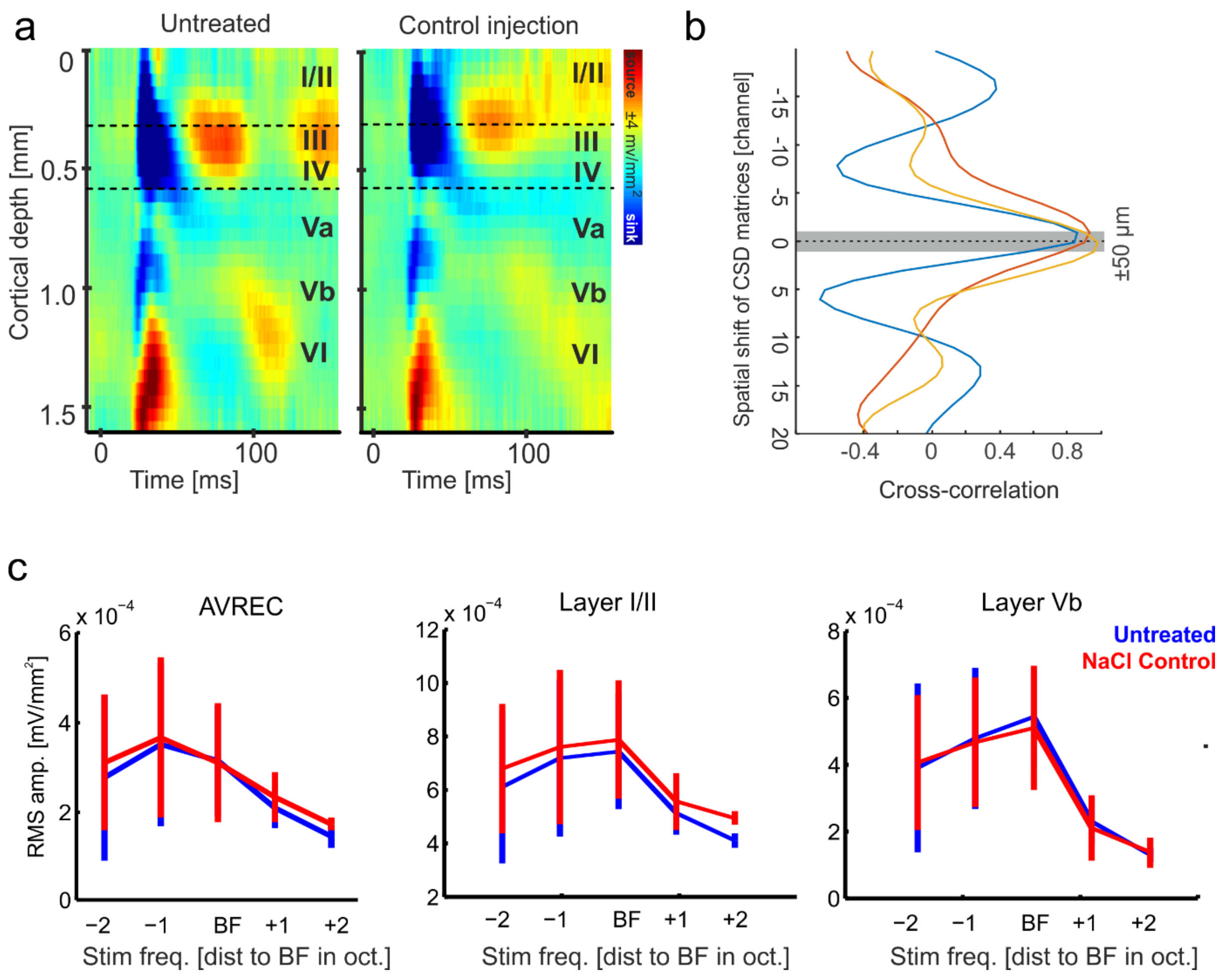

Supplemental Fig. 1. Control injection of 0.9% sodium-chloride (NaCl) did not change layer-specific processing in the auditory cortex. a Tone-evoked CSD profiles before and after control injection of 0.9% sodium-chloride displayed the canonical feedforward pattern of early and late sink activity distributed across cortical layers. Control injections did not lead to qualitative changes of tone-evoked activity. **b** In order to quantify the stability of the cortical laminae along the derivation axis and thus the comparability of the patterns before and after enzyme administration, we have cross-correlated the early onset CSD profile of each animal before and after HYase injection. Relative changes of overlapping sinks and sources of the electric field may occur, while the general spatial profile should be stable. Highest correlation should then be at a zero lag shift, while shifts of the electrode relative to cortical layers should be detectable by a shift in the peak of the cross-correlogram. Correlation peaks in our data set were all found with at 0 ± 1 channel shift corresponding to a maximal shift of ± 0.05 mm.**c** Frequency-response tuning curves of AVREC RMS amplitudes (±SEM; n=3) showed no significant change of activity across all stimulation frequencies after control NaCl injection. **c** Frequency-response tuning curves for the RMS amplitude (±SEM) of CSD traces from cortical layers I/II and Vb (which were altered after ECM removal) did also not show any significant change after control injections. Statistical significance was tested by a 2-way rmANOVA with factors ‘Freq’ and ‘Injection’. All main effects and interactions were not significant.
